# Electrochemical Measurement of Freeze-Thaw Cycle Impact on Sarcoplasmic Oxidation in Beef

**DOI:** 10.1021/acsmeasuresciau.4c00095

**Published:** 2025-04-25

**Authors:** Silan Bhandari, Sachinthani A Devage, Rishav Kumar, Ranjith Ramanathan, Sadagopan Krishnan

**Affiliations:** † Department of Chemistry, 7618 Oklahoma State University, Stillwater, Oklahoma 74078, United States; ‡ Department of Animal and Food Sciences, 7618 Oklahoma State University, Stillwater, Oklahoma 74078, United States

**Keywords:** electroanalysis, meat science, freeze−thaw, protein oxidation, redox signals, sensor

## Abstract

Repeated freezing and thawing (F-T) of meat, a common practice in home kitchens, markets, and transportation, reduces meat quality due to wide temperature fluctuations. This study presents the electrochemical analysis of oxidation in beef longissimus lumborum muscle sarcoplasm under repeated F-T cycles, which differs from prior reports that focused on other related meat aspects, such as discoloration, adulteration, freshness, and antibiotic detection. Moreover, comparing the complexity of meat extract analysis using certain spectral methods, such as Raman, NMR, FTIR, and expensive mass spectrometry, electrochemical methods offer simplicity, speed, and cost-effectiveness. Increased current responses at specific peaks (0.82 ± 0.01 V and −0.25 ± 0.01 V vs Ag/AgCl) correlated strongly (*r* = 0.99, *p* < 0.01) with elevated metmyoglobin content, which is responsible for the discoloration or brown color of meat, validated by spectrophotometry. Frozen sarcoplasm (day 3) exhibited significantly higher currents and metmyoglobin levels (*p* < 0.01) compared to fresh sarcoplasm (day 0), indicating biochemical changes during F-T cycles. Electrocatalytically accessed redox signals of purified beef myoglobin confirmed the contributions from the rapid oxidation of myoglobin, as well as other meat sarcoplasmic proteins. This research introduces a portable, cost-effective electrochemical tool for point-of-need monitoring of meat oxidation under various practical, experimental, and environmental conditions. Future research could focus on obtaining insights into biochemical changes in longissimus lumborum sarcoplasm during frozen storage and developing strategies to mitigate the effects of F-T cycles on meat quality.

## Graphical abstract

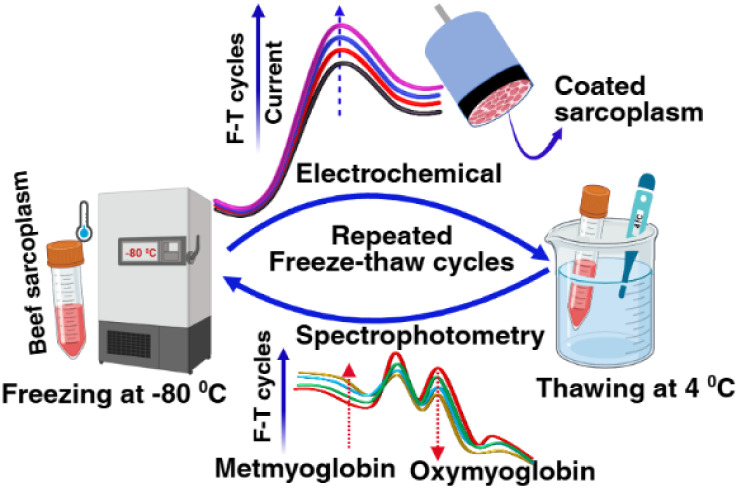

## 1 Introduction

Nutritious beef meat is a commercially valuable and highly demanded protein in the Western world.^1^ Currently, the value of meat exports worldwide is over $13 billion, and freezing is one of the methods used during export to preserve meat quality and extend its shelf life for storage.^2,3^ Repeated freezing and thawing occur in home kitchens, in the market, and during transportation during global trade.^2,4^ During freeze–thaw (F-T) cycles, large temperature fluctuations occur, which cause meat recrystallization. This leads to mechanical damage and protein and lipid oxidation, which affect meat quality, including color, texture, and water-holding capacity.^4,5^ The ice crystals formed within the muscle tissues by the freezing and thawing activity rupture the cell membrane, releasing pro-oxidants such as heme iron and lysosomal and mitochondrial enzymes that can intensify the oxidation of proteins.^6,7^ The oxidation of proteins in meat can result in diminished eating quality, as it reduces tenderness and juiciness, while also increasing discoloration.^8^ The protein and lipid oxidation process is exacerbated with the repeated F-T cycles.^9–11^ In this study, beef muscle, specifically the longissimus lumborum (LL), was used to investigate the effect of repeated freeze–thaw cycles. Due to its longer color stability (it does not turn brown quickly during retail storage), this muscle is classified as a color-stable muscle.^12,13^

Beef meat contains proteins, fats, and micronutrients such as zinc, iron, heme, and vitamin D.^14^ The heme proteins, including myoglobin, hemoglobin, and cytochromes, play a crucial role in cellular metabolism and oxygen transportation.^15^ Among these proteins, myoglobin significantly contributes to meat color. Myoglobin undergoes interconversion into three distinct redox forms: oxymyoglobin (bright red), metmyoglobin (brown), and deoxymyoglobin (purplish pink).^16^ Consumers use meat color as an indicator of its quality and freshness. Meat freshness is determined by its visual appearance, taste, hue, microbial presence, and nutritional content.^4^

Expensive, high-tech, skill-demanding instruments are not affordable, widely accessible, or user-friendly for routine point-of-need analytical applications.^17,18^ To address these limitations, our approach is based on the cost-effective and simple-to-use electrochemical sensing of the complex beef sarcoplasm of the LL muscle. We utilize electrochemistry because the various redox changes occurring in meat during repeated F-T cycles determine its quality and nutritional attributes. Previously, Raman spectroscopy was used to identify protein conformation,^17^ and real-time nuclear magnetic resonance (RT-NMR) was used to detect water movement and distribution during repeated freezing and thawing processes.^5^ Fluorescence and Fourier transform infrared (FTIR) spectroscopy were used to determine the alterations in the tertiary and secondary structures of myofibrillar proteins caused by repeated F-T cycles.^19,20^ Meat assessment using FTIR often yields complex spectra that are challenging to analyze and identify changes in under varying meat conditions. To successfully analyze meat protein and lipid adulteration in the observed meat spectrum, effective data preprocessing and chemometric methodology (i.e., multivariate statistical methods) are necessary due to the presence of undesired ambient and instrumental noise, as well as scattering effects, in the FTIR spectra.^21–25^ Furthermore, to optimize spectral interpretation, FTIR requires removing the water content of meat samples, which involves a laborious sample preparation procedure.^26^ Rapid evaporative ionization mass spectrometry (REIMS) was also used for the lipidomic profiling of fresh and frozen–thawed beef muscles.^27^ However, it is similarly constrained by complicated and expensive equipment and processing that necessitate multivariate statistical analysis.^27^

Previously, ostrich meat was differentiated from chicken, pork, and beef by coupling an electrochemical method with a chromatographic technique (HPLC-EC and LC/MS).^28^ Cyclic voltammetry was utilized to assess the freshness of beef meat by using an electrochemical signal shift from 0.06 to 0.16 V vs a Au/silicon wafer as a reference electrode during the storage period (0–9 days), without any specific biomolecule identification or validation.^29^ Furthermore, to assess the freshness of beef meat, a cyclic voltammetric approach was used to detect ammonia and putrescine.^30^ However, high background currents limit the cyclic voltammograms by decreasing the analytical charge transfer signals; therefore, they also employed a multivariate statistical method to demonstrate the usability of electrochemical sensors.^28,30^ Afterward, the ciprofloxacin antibiotic was measured in beef samples using pulse voltammetry by measuring the maximum anodic peak current (at 1.1 V vs Ag/AgCl) associated with ciprofloxacin oxidation.^31^ Additionally, an electrochemical sensor based on a locked nucleic acid probe was designed to detect the adulteration of donkey meat in beef sausage products. This electrochemical genosensor, designed for detecting donkey genetic elements in consumable beef sausages and validated by quantitative real-time polymerase chain reaction (QRT-PCR), is highly sensitive and selective, employing linear sweep, pulse voltammetry, and electrochemical impedance spectroscopy techniques.^32^

In the present report, we monitored the oxidation of beef meat and the associated changes in myoglobin redox form using the square-wave pulse technique, with a 15 min sample adsorption on the electrode and a scan duration of less than 1 min. To provide quantitative insights in conjunction with the primary focus on meat color rendering, specifically the myoglobin molecular target, we present absorbance spectra of the same samples subjected to several F-T cycles to confirm and validate the electrochemical peak current increase and redox potential signals. Our combined electrochemistry with target-specific absorbance spectroscopy enhances the scientific rigor of the methodology.

Through electrochemistry, we can determine the changes in specific oxidation and reduction peak signatures at their respective redox potentials, which correspond to the biochemical changes occurring in LL muscle, such as metmyoglobin levels (related to the brown color of meat) and other sarcoplasmic protein oxidation during repeated F-T cycles. The square wave voltammetry that we performed quickly scans [∼1 V range in a few seconds at a 375 mV/s scan rate; derived from the frequency (15 s^–1^) times amplitude (25 mV) values used] the adsorbed sarcoplasmic film on high-purity graphite surface with a high signal-to-noise ratio and diminished background charging current compared to linear sweep voltammetric techniques (e.g., cyclic voltammetry).^33,34^ The electrochemical study is combined with spectrophotometry to obtain quantitative information on the contents of metmyoglobin (MetMb) and oxymyoglobin (OxyMb) in the same sarcoplasm extracted from the LL muscle subjected to F-T cycles. Several studies indicate that repeated F-T cycles should be limited to fewer than three times, as they adversely affect meat quality when subjected to more than three cycles.^5,35,36^

The objective of this research work is to investigate the effect of multiple F-T cycles on beef LL muscles electrochemically, focusing on biochemical changes such as protein oxidation and metmyoglobin reduction, by comparing the voltammetric signals of fresh sarcoplasm (day 0) and frozen sarcoplasm (day 3) subjected to repeated F-T cycles. We also established a correlation between the increased current response of the electrochemical sensor and the elevation of metmyoglobin in beef LL muscle. Our study was conducted for up to six F-T cycles to observe the effects on the LL muscle sarcoplasm. When we compared the effect of the first and third F-T cycles in frozen LL sarcoplasm with the control sarcoplasm without F-T cycles (MetMb = 3.5%), the first F-T cycle did not have a notable impact on protein oxidation and metmyoglobin deposition (current change = 10.6 μA and MetMb = 9.1%). In comparison, the third F-T cycle showed a significant effect on the oxidation of proteins and the level of metmyoglobin (current change = 22.4 μA and MetMb = 13.31%, which is approximately four times higher than the control). The increase in relative peak current change (ΔI_p_ %) was 37.3%, and the corresponding absorbance-based estimation of metmyoglobin content was 13.2% after the sixth F-T cycle in fresh LL sarcoplasm compared to the control (without the F-T cycle). This study further demonstrates a strong positive correlation between the increasing number of F-T cycles and electrochemical peaks (*r* = 0.98) and metmyoglobin (*r* = 0.99), with the change being more significant after the third cycle. Figure 1 provides a schematic overview of the research strategy employed in this study.

**Figure 1:**
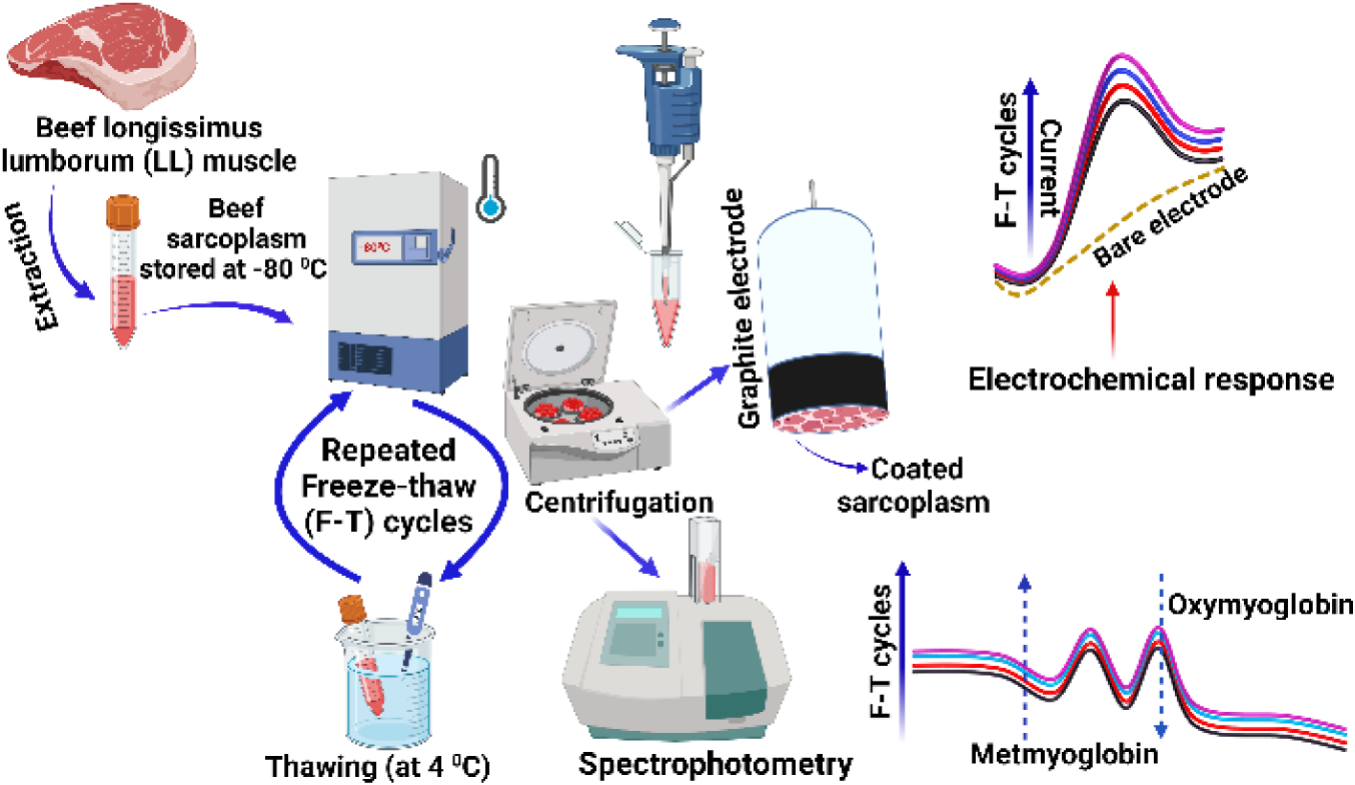
Schematic of the research strategy illustrating electrochemical and spectrophotometric measurements of beef sarcoplasm extracted from longissimus lumborum (LL) muscle subjected to multiple freeze-thaw cycles (*n* = 5 batches × 3 electrodes = 15 replicates per freeze-thaw cycle). After the freezing and thawing process, the LL muscle sarcoplasm was centrifuged, and 10 μL of the sample (pH 5.6) was coated on a polished high-purity graphite surface for voltammetric measurements. In parallel, 1.5 mL of the supernatant sarcoplasm solution was used for spectrophotometric quantitation of metmyoglobin and oxymyoglobin contents upon repeated freeze-thaw cycles.

## 2 Experimental Section

### 2.1 Reagents and Materials

High-purity graphite (HPG) electrodes, potentiostat (Model CHI 6017), silicon carbide (SiC-grit 320, Extec Corp., CT, USA), and mixed phosphate buffer (0.1 M, potassium phosphate monobasic and potassium phosphate dibasic, purchased from Sigma-Aldrich, St. Louis, MO, USA). The buffer was adjusted to a pH of 5.6 using a pH meter (Fisher Scientific, Model AB 15 Plus). Centrifugation was performed using an Eppendorf 5424 centrifuge.

### 2.2 Sample Preparation

United States Department of Agriculture (USDA) grade low-choice strip loins (*n* = 5) were collected from a commercial processing plant 14 days postmortem and transported back on ice to the Oklahoma State University Food and Agriculture Products Center. The muscles were held until 18 days postmortem. After that, the longissimus lumborum was packaged in vacuum packaging and stored in an aged room at 4 °C until 33 days postmortem.

### 2.3 Beef Sarcoplasm Extraction and Spectroscopic Determination

Sarcoplasm was extracted from the longissimus lumborum muscles of beef (*n* = 5, labeled as LL1, LL2, LL3, LL4, and LL5). Muscle samples, free from fat and connective tissue, were used for extraction.^37^ 10 g of the muscle sample was blended with 30 mL of 50 mM phosphate buffer (pH 5.6) using an Omni-Mixer (Ivan Sorvall Inc., Newtown, CT, U.S.A.) for 30 s at 4 °C. Homogenized muscle was centrifuged at 14000 × g for 5 min using an Eppendorf 5418 centrifuge (Eppendorf North America, 175 Freshwater Blvd, Enfield, CT, U.S.A.). The supernatant was passed through double-layered cheesecloth to remove visible fat and suspended particles. For further purification, the supernatant was filtered using 0.22 μm syringe filters (Merck Millipore Ltd., Tullagreen, Carrigtwohill, Ireland). Extracted sarcoplasm was measured spectrophotometrically by scanning absorption from 400 to 700 nm using a UV–vis spectrophotometer (Varian Cary 100 Bio, Varian Inc., CA). The blank solution contained only phosphate buffer, pH 5.6. The proportion of myoglobin forms was measured by the absorbance of their respective peaks in the extracted sarcoplasm solution after homogenization.^38^ Different forms of myoglobin were determined by measuring the wavelength maximum at 503 nm for metmyoglobin, 557 nm for deoxymyoglobin, and 544 and 582 nm for oxymyoglobin.^38^ The same sarcoplasmic extract of the longissimus lumborum muscles (*n* = 5) was frozen at −80 °C in a lab freezer (Thermo Fisher Scientific, Model Number: FDE60086F) for the next 72 h. Followed by a 72-h (day 3) frozen cycle, the muscle steaks were thawed in 4 °C water in a plastic beaker. After the thawing cycle, the sarcoplasmic protein was extracted, as described earlier.^37^

### 2.4 Freezing and Thawing Process

The LL beef sarcoplasm was divided into two groups. The first sarcoplasm extracted from the LL muscle was considered fresh sarcoplasm (day 0). The same LL muscles were frozen for 3 days (at −80 °C), and the extracted sarcoplasm from them was considered frozen sarcoplasm. The freshly extracted sarcoplasm from beef LL muscle (*n* = 5) and 3 days of frozen sarcoplasm from the same LL muscle (*n* = 5, LL1 to LL5) were each subjected to multiple F-T cycles. These repeated F-T cycles were conducted with freezing at −80 °C and thawing at 4 °C cold water in a beaker, respectively. After each F-T cycle, the sarcoplasm of the LL muscle was examined electrochemically by scanning the positive and negative potential regions using freshly made sample electrodes for each region. The spectrophotometry validation was performed in parallel with the electrochemical measurements for each sample. A beef sarcoplasm sample not subjected to freezing and thawing was used as a reference sample for comparison.

### 2.5 Electrochemical Measurements

The electrochemical study was performed under saturated oxygen conditions at 37 °C by using a CHI potentiostat (Model CHI 6017) and a standard 3-electrode electrochemical cell featuring an Ag/AgCl (1 M KCl) reference electrode, a Pt-wire counter electrode, and polished high-purity graphite disk (HPG, geometric area 0.316 cm^2^) as working electrodes coated with sarcoplasmic extract samples or with purified beef myoglobin.

Before coating the meat extract or purified myoglobin on HPG electrodes, the electrodes were polished on SiC-grit 320, sonicated for 30 s in water, and dried under ultrapure nitrogen. An extract solution (10 μL, pH 5.6) was applied to the polished HPG electrodes and dried in air at room temperature (23 °C, 15 min duration), producing a meat extract biofilm coating that allowed for rapid electrochemical measurement within a few seconds. The purified beef myoglobin solution (*n* = 3, 1.3 mg/mL, pH 5.6) was prepared using the same method for sarcoplasm extraction.^37^10 μL of this solution (without F-T cycles) was applied to an electrode to obtain the voltammogram peaks and relate them to the meat extract data. Highly sensitive pulse square wave voltammetry was performed in a 10 mL phosphate buffer (pH 5.6, 37 °C) over two distinct potential ranges using new sample electrodes for each range, from −0.1 to +1.2 V and from 0.1 to −0.7 V vs an Ag/AgCl reference electrode (1 M KCl). The parameters for the square wave voltammetry were a 15 Hz frequency, a 25 mV pulse height, and a 4 mV step height.

## 3 Results and Discussion

The square wave voltammograms were examined in an oxygenated buffer solution (pH 5.6 at 37 °C) using sarcoplasm extract-coated polished HPG disk electrodes. Due to their exceptional electrochemical properties, HPG electrodes are effective in electrochemical studies, substituting for glassy carbon in other carbon-based electrodes.^39^ Polished HPG electrodes without a coated sarcoplasm extract did not show any peaks in the scanned potential regions, confirming the usefulness of HPG electrodes in monitoring beef LL sarcoplasmic extract-based electrochemical signals, as shown in Figure 2. The square wave voltammograms of meat extract-coated or purified beef myoglobin-coated electrodes revealed peaks at approximately +0.82 ± 0.01 V vs Ag/AgCl in the positive potential range and −0.25 ± 0.01 V vs Ag/AgCl in the negative potential range, as shown in Figures 2A–F and S1. The LL sarcoplasm, without any F-T cycle treatment, was used as the control to assess the current response when the LL sarcoplasm was subjected to repeated F-T cycles. The control LL sarcoplasm displayed a smaller peak current (Ip) intensity compared to the F-T cycled sarcoplasm film on electrodes, as shown in Figures 2G,H and S2A. The increased current response on day 3 of frozen LL sarcoplasm is attributed to the frozen storage conditions, which create a favorable environment for protein degradation in LL muscles.^40–42^

**Figure 2:**
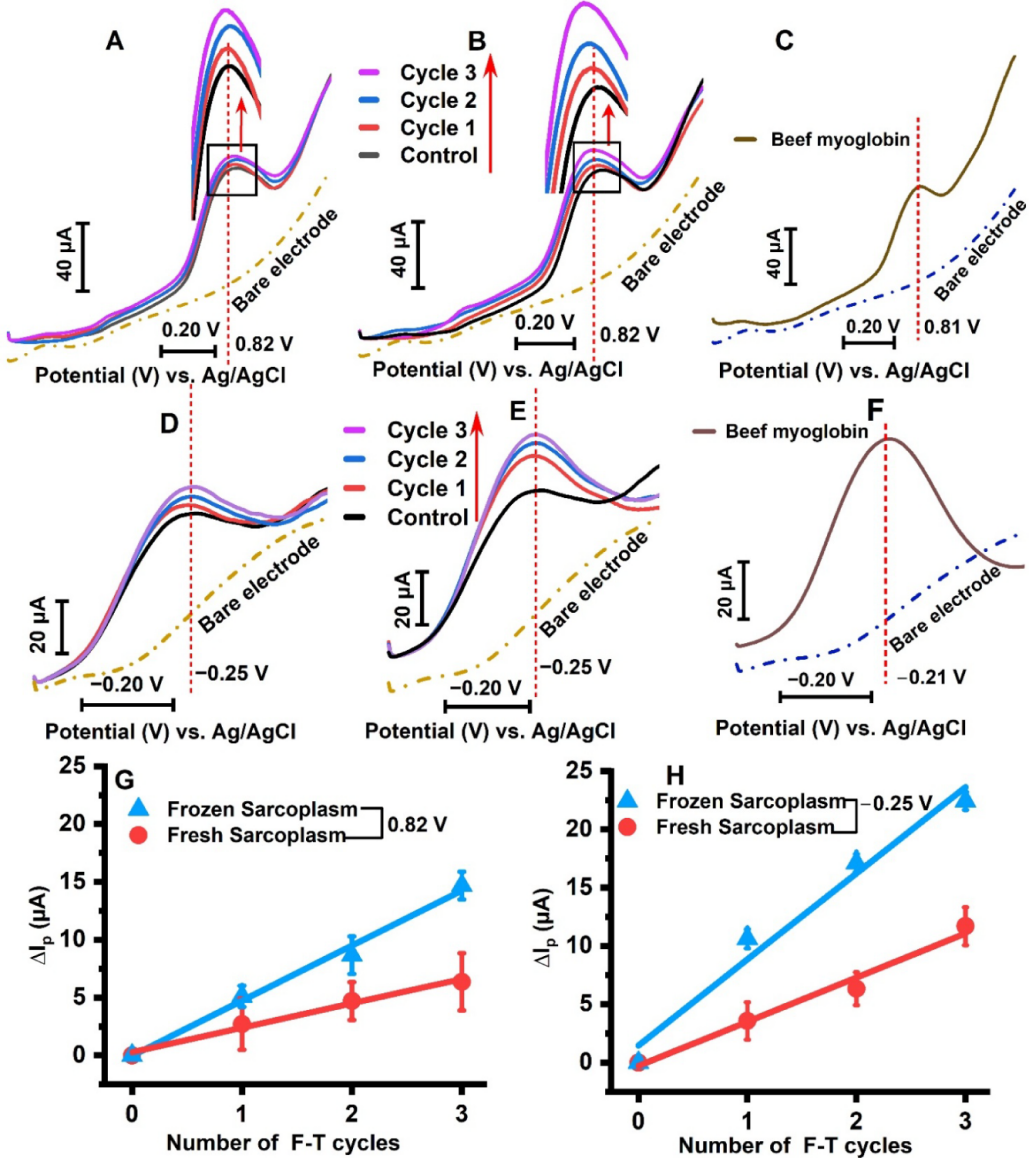
Electrochemical peak changes on beef sarcoplasm extract-coated electrodes with increased F-T cycles. The positive potential range with repeated F-T cycles for (A) Fresh sarcoplasm and (B) Frozen sarcoplasm at 0.82 ± 0.01 V vs the Ag/AgCl reference at pH 5.6. (C) The voltammogram of purified beef myoglobin (1.3 mg/mL, pH 5.6, *n* = 3 batches × 3 electrodes = 9 replicates) coated electrode without F-T cycles shows a similar peak potential (at 0.81 ± 0.01 V vs Ag/AgCl) as that of beef sarcoplasm measured in the positive potential range. The negative potential range peaks under repeated F-T cycles for (D) Fresh sarcoplasm and (E) Frozen sarcoplasm at −0.25 ± 0.01 V vs the Ag/AgCl reference at pH 5.6. (F) The voltammogram of the purified beef myoglobin-coated electrode, without F-T cycles, shows a peak potential (at −0.21 ± 0.01 V vs Ag/AgCl) that is slightly more positive, likely due to the absence of all other biological components, as in the meat extract. This is similar to that of beef sarcoplasm in the negative potential region. (G) The peak current change (ΔI_p_) measured with respect to the control sample (fresh sarcoplasm without F-T cycles) reflects an increase in current value with an increasing number of F-T cycles. The extent of current change (ΔIp) is higher in frozen sarcoplasm than in fresh sarcoplasm in the positive potential region at +0.82 V vs Ag/AgCl and (H) in the negative potential region at −0.25 ± 0.01 V vs Ag/AgCl. The parameters for the square wave voltammetry are a 15 Hz frequency, a 4-mV step height, and a 25-mV pulse height. (*n* = 5 batches of samples x 3 electrodes = 15 replicates/freeze–thaw cycle).

During the repeated F-T cycles, the positive potential scan revealed the oxidation of sarcoplasmic proteins, as indicated by oxidation peaks. In contrast, the negative potential in an oxygen environment facilitated the electrocatalytic reduction of oxygen, a characteristic of heme proteins such as myoglobin, present in the extract, via direct electron transfer with the graphite surface.^34,43–45^ This observation confirmed the role of myoglobin and its oxidation in the complex beef sarcoplasm treated under repeated F-T cycles. The spectrophotometric measurement was performed in parallel with the electrochemical study using the same samples. The wavelength peaks were reported to be at 503 and 632 nm for metmyoglobin, 557 nm for deoxymyoglobin, and 544 and 582 nm for oxymyoglobin.^38^ In agreement, the wavelengths of maximal absorption were observed at 503, 557, and 582 nm for metmyoglobin, deoxymyoglobin, and oxymyoglobin, respectively, as shown in Figures 3A–D, S2B and S3. The number of repeated F-T cycles strongly correlated with an increase in metmyoglobin content (*r* = 0.98 for fresh and *r* = 0.96 for frozen sarcoplasm) and a decrease in oxymyoglobin content (*r* = −0.86 for fresh and *r* = −0.97 for frozen sarcoplasm), as shown in Figures 3C,D and S2B.

**Figure 3:**
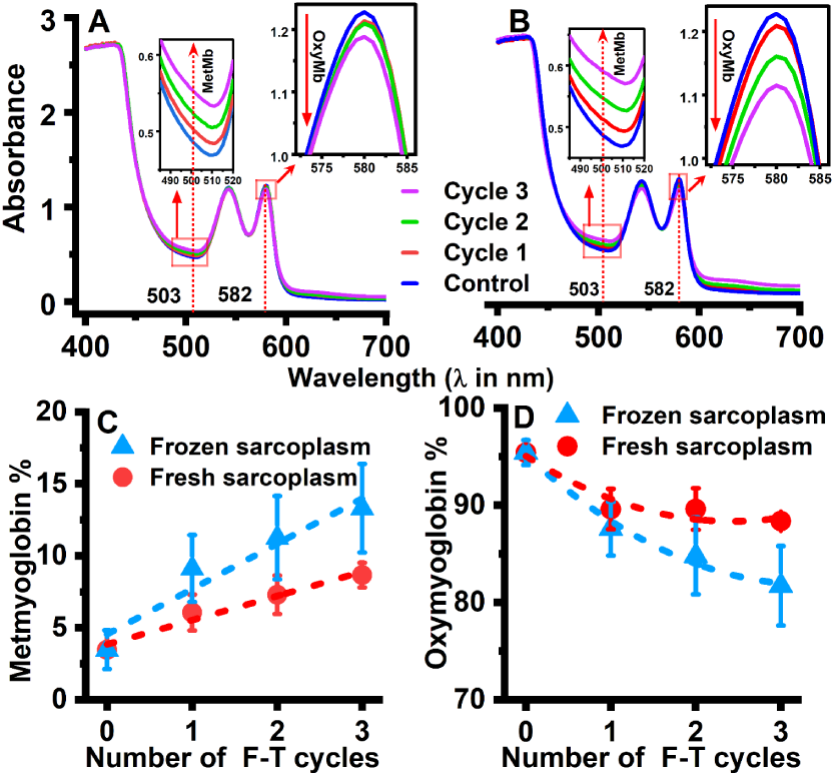
UV–visible spectrophotometry of beef LL muscle sarcoplasm extract solution was performed in standard UV–vis cuvettes (Dimensions: 12.5 mm × 12.5 mm × 45 mm, 1.5 mL extract, pH 5.6, *n* = 5 samples with three replicates) under repeated F-T cycles treatment. Measurements were taken with pH 5.6 phosphate buffer as the blank for baseline correction. The absorbance of oxymyoglobin at 544 and 582 nm and metmyoglobin at 503 nm is shown. (A) Fresh sarcoplasm and (B) Frozen sarcoplasm solutions. The spectrophotometric comparative plot of the increase in metmyoglobin content and decrease in oxymyoglobin content between fresh and frozen sarcoplasm solutions is shown in Figures C and D, respectively.

We observed a significant difference in metmyoglobin (*p* < 0.01) and oxymyoglobin (*p* < 0.05) quantities between fresh and frozen sarcoplasm, as shown in Figure 3A–D. The positive region peak current measured from voltammograms is attributed to the overall meat oxidation, including the metmyoglobin treated under repeated F-T cycles. The negative region peak current, which characterizes metmyoglobin-driven oxygen reduction, also showed a strong correlation with the metmyoglobin content in both fresh and frozen sarcoplasm (*r* = 0.99, *p* < 0.01) under repeated F-T cycles. Furthermore, a strong positive correlation (*r* = 0.95) was observed between the peak current and absorbance value for metmyoglobin at 503 nm in fresh LL sarcoplasm treated under six F-T cycles, as shown in Figure S4. The correlation between metmyoglobin and oxymyoglobin, as determined by spectrophotometry, and oxidative and reductive currents measured during repeated F-T cycles, indicates that our proposed electrochemical technique can detect protein oxidation, including the major meat color-rendering myoglobin redox form changes that occur in LL muscle sarcoplasm during the repeated F-T process.

## 4 Conclusions

In this study, the extent of oxidation of proteins in the positive potential region and the electrocatalytic oxygen reduction characteristics of heme proteins, such as myoglobin, in the negative potential region were investigated electrochemically using beef LL muscle sarcoplasm extract-coated electrodes under a repeated process of freezing and thawing. We used control sarcoplasm (without F-T cycles) to delineate the effect of rapid oxidation of myoglobin and other sarcoplasmic proteins resulting from repeated F-T cycles in the LL muscle sarcoplasm. The purified myoglobin and spectrophotometry studies validate the contribution of myoglobin to the redox signals measured in the LL muscle sarcoplasm. The strong correlation (*r* = 0.99, *p* < 0.01) between the electrochemical measurements in both positive and negative potential regions and the spectra suggested a noticeable effect of repeated F-T cycles on the oxidation of LL sarcoplasmic proteins.^4,5^ For the first time, the electrochemical approach is used to study the effect of freeze-thaw cycles on beef meat. This study could be significant in developing portable point-of-need electrochemical devices to investigate the properties of meat exposed to various practical, experimental, and environmental conditions relevant to the USDA’s mission of improving food quality through novel technologies and minimizing food loss and waste.

## Supplementary Material



## Notes

Figure S1: Voltammograms with increased F-T cycles (0–6 cycles) at −0.24 ± 0.02 V vs Ag/AgCl. Figure S2: Relative peak current change (% ΔI_p_) with an increased number of F-T (0 to 6) cycles and spectrophotometric determination of metmyoglobin and oxymyoglobin percentages. Figure S3: UV–visible spectrophotometry analysis of the 544 nm peak. Figure S4: A correlation plot of peak current vs absorbance (PDF)
