# Supplementary material for: Electrochemical Measurement of Freeze-Thaw Cycle Impact on Sarcoplasmic Oxidation in Beef
Source: ACS Meas Sci Au. 2025 Apr 25;5(3):287–93. doi: 10.1021/acsmeasuresciau.4c00095 (PMC12183597; doi:10.1021/acsmeasuresciau.4c00095)
Supplement: Supplementary file 1 [file tg4c00095_si_001.pdf]

Supporting Information

Electrochemical Measurement of Freeze-Thaw Cycle Impact on Sarcoplasmic Oxidation in Beef

Silan Bhandari,[a] Sachinthani A. Devage,[a] Rishav Kumar,[b] Ranjith Ramanathan*,[b] Sadagopan Krishnan*,[a]

[a]Department of Chemistry, Oklahoma State University, Stillwater, OK,74078, USA

[b]Department of Animal and Food Sciences, Oklahoma State University, Stillwater, OK, 74078, USA

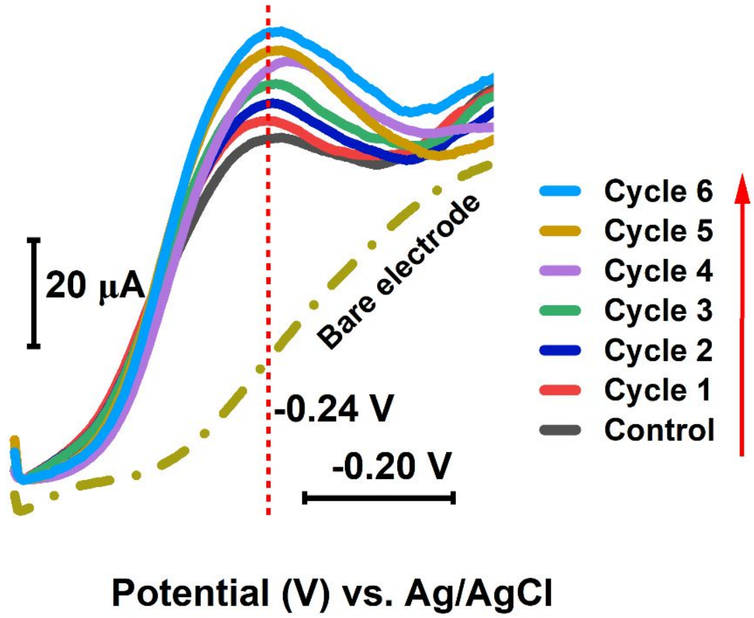

Figure S1. The electrochemical peak from fresh LL beef sarcoplasm extract coated electrodes with increased F-T cycles (0-6 cycles) at -0.24 ± 0.02 V vs. Ag/AgCl in the negative potential range showing increasing peak current with increasing number of F-T cycles.

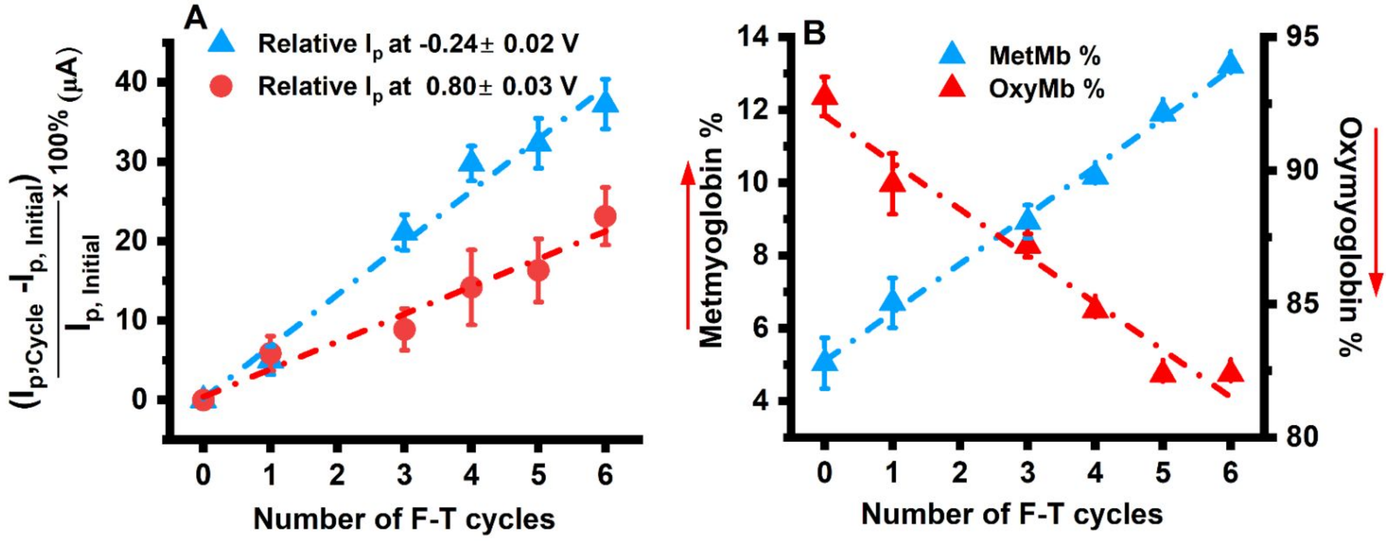

Figure S2. (A) Relative peak current change (ΔI_p %) with an increased number of F-T (0 to 6) cycles for fresh LL muscle sarcoplasm in both positive and negative potential regions. (B) Spectrophotometric determination of metmyoglobin and oxymyoglobin percentages with an increased number of F-T (0 to 6) cycles (n=3, phosphate buffer, pH 5.6).

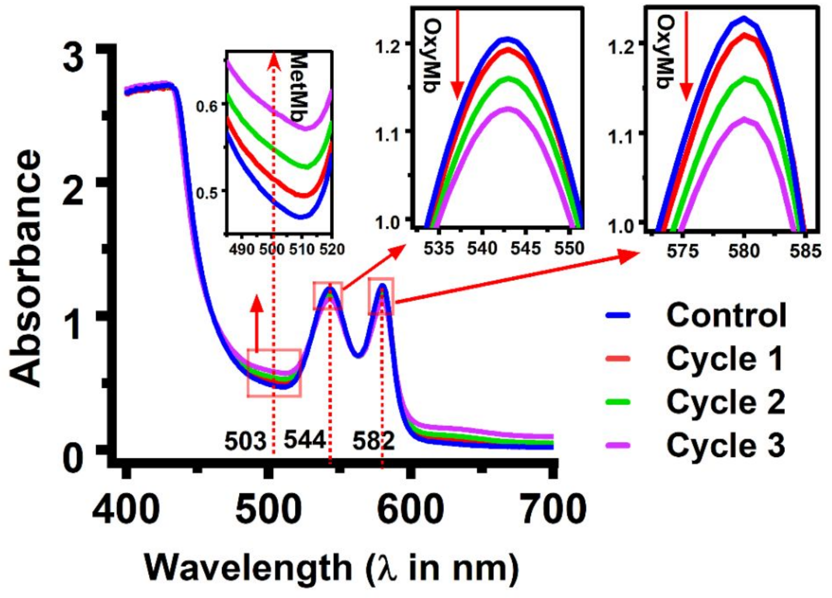

Figure S3. UV-visible spectrophotometry of beef LL muscle sarcoplasm extract solution under repeated F-T cycles showing the absorbance of metmyoglobin at 503 nm and oxymyoglobin at 544 and 582 nm.

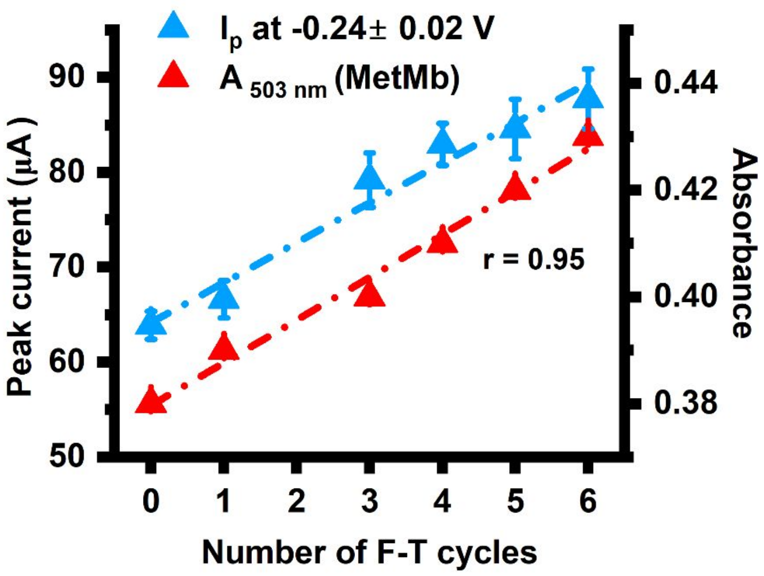

Figure S4. A correlation plot of peak current vs. absorbance (at 503 nm for metmyoglobin, denoted as MetMb) shows a strong positive correlation (r= 0.95) for fresh sarcoplasm.
